# A framework to understand the role of biological time in responses to fluctuating climate drivers

**DOI:** 10.1038/s41598-022-13603-5

**Published:** 2022-06-21

**Authors:** Luis Giménez, Noé Espinosa, Gabriela Torres

**Affiliations:** 1 Biologische Anstalt Helgoland, Alfred-Wegener-Institut, Helmholtz-Zentrum Für Polar- Und Meeresforschung, 27498 Helgoland, Germany; 2 School of Ocean Sciences, Bangor University, Menai Bridge, LL59 5AB UK

**Keywords:** Ecology, Evolution, Physiology

## Abstract

Understanding biological responses to environmental fluctuations (e.g. heatwaves) is a critical goal in ecology. Biological responses (e.g. survival) are usually measured with respect to different time reference frames, i.e. at specific chronological times (e.g. at specific dates) or biological times (e.g. at reproduction). Measuring responses on the biological frame is central to understand how environmental fluctuation modifies fitness and population persistence. We use a framework, based on partial differential equations (PDEs) to explore how responses to the time scale and magnitude of fluctuations in environmental variables (= drivers) depend on the choice of reference frame. The PDEs and simulations enabled us to identify different components, responsible for the phenological and eco-physiological effects of each driver on the response. The PDEs also highlight the conditions when the choice of reference frame affects the sensitivity of the response to a driver and the type of join effect of two drivers (additive or interactive) on the response. Experiments highlighted the importance of studying how environmental fluctuations affect biological time keeping mechanisms, to develop mechanistic models. Our main result, that the effect of the environmental fluctuations on the response depends on the scale used to measure time, applies to both field and laboratory conditions. In addition, our approach, applied to experimental conditions, can helps us quantify how biological time mediates the response of organisms to environmental fluctuations.

## Introduction

One of the biggest challenges faced by humanity is climate change^1–3^. A key characteristic of current climate change is the presence of extreme climatic events, i.e. strong fluctuating environmental conditions, manifested as storms, hurricanes and heatwaves. Hence, a challenge for ecologists consists in quantifying and predicting the responses of ecological systems (from populations to ecosystems) to such fluctuations^4–7^. Despite the emphasis in understanding ecology and evolution in fluctuating environments^8–11^ most experiments concerning climate driven environmental variables focus on responses to constant conditions^11–13^. The currently growing body of work tackling responses to fluctuating environments highlights the complications in attempting to incorporate the role of variation in such environmental drivers^13–17^ especially in dealing with their combined actions^18,19^. For instance, research on extreme events has identified five primary traits characterising heatwaves^20^, which should be added to the effect produced by the average condition experienced during that event.

An additional layer of complexity is given by the biological time (e.g. time to metamorphosis, to reproduction, generation times), characterising biological systems^21^. Biological time is governed by the interaction between the environmental and physiological processes and plays a critical role in driving fitness, population dynamics and community structure^21–25^. Biological time is critical because of three reasons. First, responses (e.g. survival rates) to environmental conditions are driven by a number of developmental processes operating at different biological time scales^26–31^, ranging from short (e.g. physiological acclimation) to medium (developmental plasticity) and long term (transgenerational plasticity, changes in gene frequencies). Second, if we characterise a fluctuation by its time scale, then the response will depend on the biological time characterising that species. From the perspective of organisms (e.g. with time scales ranging from those of from bacteria to trees), whether a fluctuation is long or short, is not determined by the chronological (= clock) time, but instead by its characteristic biological time^31^. Third, responses may be measured after a predetermined chronological time scale (e.g. at a given time in the year) or biological time scale (e.g. at maturity). The expression of biological responses in chronological time is obviously important to understand long term changes in seasonal habitats. However, measurements on biological time scales are critical for understanding population dynamics and evolution because such number drives individual fitness and the population growth rate. This is especially important in species that experience habitat shifts (e.g. most bottom living marine invertebrates, insects, migratory fish, birds and mammals). In such species, the biological time is “reset” at critical stages (e.g. metamorphosis, onset of migration) because, after the habitat shift, organisms experience the environmental conditions of a new habitat and the conditions in the old habitat might become irrelevant. Overall, understanding and quantifying the actual response to climate driven fluctuations requires that we also understand how responses are modified with a change in the time reference frame (from chronological to biological).

**Figure 1:**
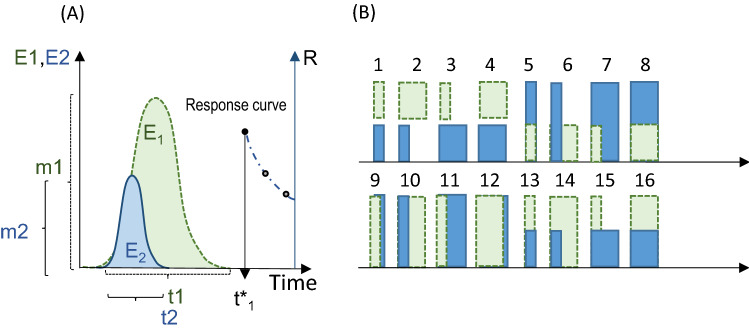
Experimental set up and equations. (**A**) A biological system (e.g. an organism) is exposed to two fluctuating environmental drivers (*E*_*1*_*, E*_*2*_). The fluctuations are characterised by predictors, i.e. the amplitudes (*m1, m2*) and time scales (*t*_*1*_*, t*_*2*_). Measurement of the response, *R*, are made at different times ($t{^*}$_1_) after the fluctuations occurred (black circle); additional measurements may be carried out at other fixed times after $t{^*}$_1_ (grey circles). (**B**) In a factorial experiment the process would be repeated so that observations are made for a minimum of two levels per predictor giving 16 factor combinations. In (**B**): vertical dimension = magnitude; horizonal dimension = clock time scale.

We propose a framework to understand and quantify responses to fluctuations in one or more climate-driven environmental variables, considering biological time. We consider environmental fluctuations characterised by a magnitude (e.g. the amplitude) and a timescale (e.g. the period of the fluctuation or the time of exposure to a given magnitude: Fig. 1). The response is quantified at least once after the environmental drivers are experienced, with respect to chronological time or at a given life history event (e.g. at maturity). We use an experimental case and simulations to understand how biological responses to fluctuating environmental drivers are modified by the clock and biological time scale used to study the response. We structure this article as follows: First, we introduce definitions and a system of equations describing the biological responses. Second, the equations are explored using four specific cases. Third, in the methods section, we describe the experiments carried out to illustrate case 3.

## Results

### Mathematical theory

We consider a biological response (e.g. body size, survival, biodiversity) to two environmental drivers (i.e. any abiotic or biotic factor) but the same idea may be applied to a larger number of drivers. The response depends of a set of predictors consisting in the magnitudes (*m*_1_ and *m*_2_) and time scales of fluctuation of two drivers (*i* = 1, 2); in addition, the response is quantified at least once after the fluctuations have been experienced (Fig. 1a).

Time is defined using two different frames; chronological (= clock) time (measured by clocks) and biological time. For the “clock” time scales of the fluctuations (*t*_1_*, t*_2_) there are associated biological times (*τ*_1_*, τ*_2_). Likewise, for the clock time at which the response is quantified (${t}^{^*}$) there is an associated biological time (*τ*${^*}$).

Biological time is the proportion of (clock) time needed to reach a life history event (e.g. moulting, maturity). Hence, for *t*_*1*_*, t*_*2*_ and *t*${^*}$ we obtain *τ*_*i*_ = *t*_*i*_*/D* and *τ*${^*}$ = *t*${^*}$*/D,* (D = clock time needed to reach such life history event). We express the *τ*_*i*_ and *τ*${^*}$ in terms of a function *L* = *1/D*. For instance, for *t*${^*}$ we obtain:

1 $${\tau }^{^*}={t}^{^*}\cdot{L}$$

where *L* = *L*(***ω***) = *D*^−1^(***ω***) characterises the timing of a life history event (with units as the inverse of clock time units). *L* depends on the set of predictors ***ω*** associated to the fluctuations; an important set of predictors will be defined by thermal fluctuations (the amplitude and time scales), which in ectotherm species have a strong influence on developmental time^32,33^. We find by differentiation that *L* provides the transform function between clock and biological time frames; for instance, if *L* does not depend on any *t*_*i*_ we have *L* = *dτ/dt*_*i*_*.*

The response is expressed as a function of the predictors defined above, as *R*(*m*_1,_* m*_2,_* t*_1_,* t*_2,_* t*${^*}$) = *r*[*m*_1,_ *m*_2,_ *τ*_1_ (*t*_1_),* τ*_*2*_(*t*_*2*_)_,_ *τ*${^*}$]. The contribution of each predictor to the response is better understood by the partial derivatives with respect to each predictor; this defines a system of partial differential equations (PDE; Supplementary note 1) which expressed in matrix form give the following matrix equation.

2 $$\left[\begin{array}{c}\frac{dR}{d{m}_{1}}\\ \frac{dR}{d{m}_{2}}\\ \frac{dR}{d{t}_{1}}\\ \frac{dR}{d{t}_{2}}\\ \frac{dR}{d{t}^{^*}}\end{array}\right]=\left[\begin{array}{ccccc}1& \frac{d{m}_{2}}{d{m}_{1}}& \frac{d{\tau }_{1}}{d{m}_{1}}& \frac{d{\tau }_{2}}{d{m}_{1}}& \frac{d{\tau }^{^*}}{d{m}_{1}}\\ \frac{d{m}_{1}}{d{m}_{2}}& 1& \frac{d{\tau }_{1}}{d{m}_{2}}& \frac{d{\tau }_{2}}{d{m}_{2}}& \frac{d{\tau }^{^*}}{d{m}_{2}}\\ \frac{d{m}_{1}}{d{t}_{1}}& \frac{d{m}_{2}}{d{t}_{1}}& \frac{d{\tau }_{1}}{d{t}_{1}}& \frac{d{\tau }_{2}}{d{t}_{1}}& 0\\ \frac{d{m}_{1}}{d{t}_{2}}& \frac{d{m}_{2}}{d{t}_{2}}& \frac{d{\tau }_{1}}{d{t}_{2}}& \frac{d{\tau }_{2}}{d{t}_{2}}& 0\\ 0& 0& 0& 0& \frac{d{\tau }^{^*}}{d{t}^{^*}}\end{array}\right]\cdot \left[\begin{array}{c}\frac{dr}{d{m}_{1}}\\ \frac{dr}{d{m}_{2}}\\ \frac{dr}{d{\tau }_{1}}\\ \frac{dr}{d{\tau }_{2}}\\ \frac{dr}{d{\tau }^{^*}}\end{array}\right]$$

In the PDE (Eq. 2), the left-hand side is a vector column of the derivatives of the response in clock time (**R**), with respect to each predictor; the right-hand side is the standard (= inner) product of a matrix (**M**) by a vector of the derivatives of the response in biological time (**r**), i.e. **R = Mr**. The matrix contains the derivatives of the predictors with respect to each other, with time both expressed in clock or biological scales; one can think of **M** as an object containing coefficients that transform **r** into **R** in the same way as a constant (= 1000) would transform kilometres into meters of distance. The large number of terms in **M** highlights the considerable diversity and the challenges in quantifying responses to multivariate environmental fluctuations. We show below how to use Eq. (2) to quantify the effect of fluctuating environmental drivers on biological responses, as mediated by biological time.

First, we note that **M** contains three groups of terms: (1) Terms accounting for situations where the magnitude of a driver affects the magnitude of the second driver (e.g. temperature drives oxygen concentration in aquatic habitats): these are *dm*_*i*_*/dm*_*j*_ for any *i, j* = 1, 2. (2) Terms accounting for cases where the magnitudes and time scales of stressors are related: *dm*_*i*_*/dt*_*j*_ and *dm*_*i*_*/dt*_*i*_*.* (3) Terms where biological time depends on the magnitude or time scale of the environmental fluctuation *dτ*_*i*_*/dt*_*j*_ and *dτ*_*i*_*/dm*_*j*_. The terms of groups (1) and (2) are zero when they are mutually independent, such as in a factorial experiment with orthogonal manipulation. We will set those to zero in the rest of this analysis.

Second, we note that for group (3) there are three scenarios: (3a) biological time does not depend on any environmental driver. This is the trivial case where biological time is proportional to clock time, not considered here; **M** is simplified to a diagonal matrix, i.e. with constants in the diagonal, and zero’s otherwise leading to a single constant term per equation (3b). Biological time depends on the magnitudes of any or both drivers. In such case, *τ*_*1*_* τ*_*2*_*, and τ*${^*}$ will be driven by the same equation: if *τ*_*i*_ = *t*_*i*_* · L *(*m*_1_,* m*_2_) we obtain *dτ*_*i*_*/dt*_*j*_ = *dτ*_*i*_*/dt*_*i*_ = *L *(*m*_1_,* m*_*2*_)*.* (3c) Biological time depends on the time scale of the fluctuations: in such case, differentiating Eq. (1) with respect to time, we obtain *dτ*_*i*_*/dt*_*i*_ = *L* + *t*_*i*_* dL/dt*_*i*_.

Here, we explore four special cases where the equations are simplified to highlight the importance of biological time in modifying the responses as compared to clock time. We start with the simplest case where there is a single environmental variable and then we consider cases with two variables. We focus on cases representing the most frequent experiments carried out on multiple driver research, i.e. factorial manipulations where terms of the groups 1 and 2 are zero.

### Case 1: responses to the magnitude of a single variable

We start with the simplest case i.e. where the response is driven by the magnitude of a single driver, e.g. temperature (= m). Examples of this case are laboratory experiments quantifying the effect of temperature on body mass or survival of a given species, or mesocosm experiments quantifying effects of temperature on species richness where thermal treatments are kept constant over time. Here, the response is quantified at different times, both in the clock and biological frames. In such case we have *R*(*m, t*${^*}$) = *r*[*m, τ*${^*}$(*m, t*${^*}$)] and the PDEs simplify to.

3 $$\frac{dR}{dm}=\frac{\partial r}{\partial m}+\frac{\partial r}{\partial {\tau }^{^*}}\cdot \frac{d{\tau }^{^*}}{dm}$$

**Figure 2:**
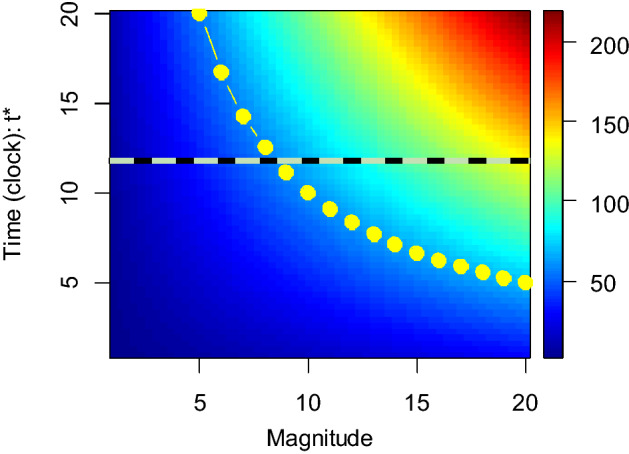
Case 1: Response to the magnitude of a single variable (*m*)*.* Horizontal line: measurement taken at clock time *t*${^*}$ = *t*${^*}$_*c*_; note that, along the line, the response increase with *m* (it crosses the colour gradient). Curve with yellow circles: measurements taken at a constant biological time (*τ*${^*}$*c* = *100*)*;* along the curve, the response does not vary with *m*. The equations used were: *R* = *m*(*0.5t*${^*}$), * τ*${^*}$ = *t. m* giving *r* = *0.5. τ*${^*}$ not depending on *m*.

From Eq. (3), and because *dR/dm ≠ dr/dm*, we see that the response to the magnitude of the driver depends on a component quantifying the effect biological time: as long as *dτ*${^*}$*/dm ≠ 0* the time reference frame affects the observed effect of *m* on the response. The simulation illustrated in Fig. 2 shows a case where there are differences between the observed responses at clock vs biological times. In the simulated experiment, there is a strong effect of the magnitude of the driver on the response at clock time, but such effect is much less pronounced at biological time. By contrast, there is no effect when the response is measured in the biological time frame.

**Figure 3:**
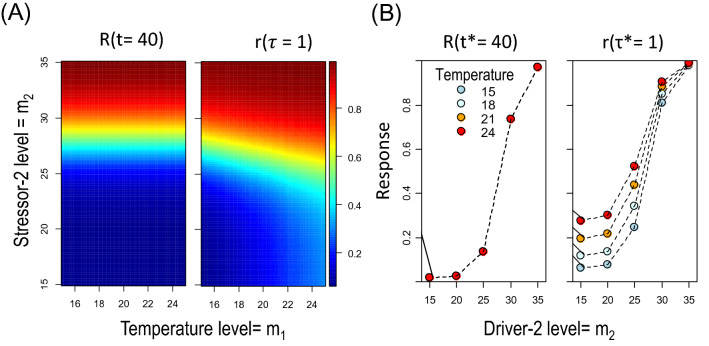
Case 2: Multiple driver responses. (**A**) Modelled responses (colour scale) at a specific clock (*t*${^*}$ = *40*) and biological times (*τ*${^*}$ = *1*), showing an interactive effect only in the biological time frame. (**B**) Interaction plots of the responses for specific levels of temperature and a second driver showing that the effect high temperature mitigates the negative effect of the second driver on the response. The response was modelled with as a sigmoidal function *R* = *exp(−t*${^*}$*φ*) with *φ* = 0.1[1 + *exp*(*m*_*2*_*/2*)]^*−*1^ to produce a strong gradient in the range of m_2_ = 25–30 units. The biological time was modelled based on the effect of temperature on the development of marine organisms^33^ as so that *t*${^*}$ = *τ*${^*}$ *exp*[*−*22.47 + 0.64/(*k(m*_*1*_ + 273)], i.e., using the Arrhenius equation with k: Boltzmann constant (≈ 8.617 10^–5^ eV K^−1^).

Equation (3) (details in Supplementary code 1) captures an obvious but important feature of experiments manipulating temperature over the development of ectotherms, for instance, from birth to metamorphosis; namely that there is no consistent definition of a simultaneous event across the different time frames. Experiments are usually stopped at different clock times because organisms must be sampled at the same biological time. All points located in the horizonal line in Fig. 3 represent simultaneous events, as defined in clock time occurring at different temperatures (e.g. whether an animal is dead or alive); however, simultaneous events occurring in biological time are represented by the points on the curve. Hence, Fig. 2 gives a geometric representation of such fact. Temperature as a driver of developmental rates^32^ is a central candidate to produce responses that differ at clock vs biological time.

We explore further this case with an example where the response is expressed as a function of time and an instantaneous rate *μ*(*m*) quantifying for instance mortality, growth or biomass loss. For this example, we obtain *R*(*m, t*${^*}$) = *r*[*μ*(*m*),* τ*${^*}$(*m, t*${^*}$)]. By differentiating in both sides, we get:

4 $$\frac{dR}{dm}=\frac{\partial r}{\partial \mu }\cdot \frac{d\mu }{dm}+\frac{\partial r}{\partial {\tau }^{^*}}\cdot \frac{d{\tau }^{^*}}{dm}$$

Equation (4) shows that *m* affects the response through two components: the instantaneous rate (*dμ/dm)* and the biological time (*dτ*${^*}$*/dm*). We call the first component “eco-physiological” and the second component “phenological” (*m* drives the timing of a biological event, e.g. time to maturation). Those components are not evident if the response is expressed in clock time; otherwise we would obtain *dR/dm* = *∂R/∂μ · dμ/dm*.

In order to better understand Eq. (4), consider an example where the response is biomass loss experienced by an organism during the process of migration (e.g. towards a feeding or reproductive ground); when the access to food during migration is very limited the result should be a decrease in body mass reserves through time. Let biomass (*B*) be modelled as an exponential decaying function of time and an instantaneous rate of biomass loss *μ*; let *μ* depend on temperature (= m) such that, *μ* = *μ(m).* In such case we obtain:

5 $$B(m,t)={e}^{-\mu \left(m\right)\cdot {t}^{^*}}={e}^{-\mu \left(m\right)\cdot {\tau }^{^*}\left(m,{t}^{^*}\right)}$$

By differentiation in both sides of Eq. (5) we get:

6 $$\frac{dB}{dm}={-e}^{-\mu \left(m\right)\cdot {\tau }^{^*}\left(m,{t}^{^*}\right)}\left\{{\tau }^{^*}\cdot \frac{d\mu }{dm}+\mu \cdot \frac{d{\tau }^{^*}}{dm}\right\}$$

Equation (6) shows the eco-physiological (*dμ/dm*) and phenological components (*dτ*${^*}$*/dm*) within the brackets. If *μ* responds linearly to temperature, then *dμ/dm* would be represented by a constant quantifying the thermal sensitivity of biomass loss; the value of such constant would depend on physiological processes associated to use of reserves to sustain movement and the basal metabolic rate. Likewise, if *τ*${^*}$ responds linearly to temperature, the *dτ*${^*}$*/dm* would be driven by a constant controlling the sensitivity of developmental time to temperature.

Because biomass is a trait that is central to fitness, Eq. (6) gives the indirect contribution of phenological and physiological responses to fitness. Assuming that fitness should be maximised, adaptive responses should involve the mitigation of negative effect of *m* on both components of Eq. (5), represented by the partial derivative of the right-hand term. For instance, organisms with the ability to minimise the eco-physiological effect (through e.g. a compensatory physiological mechanisms) or the phenological effect (e.g. shortening the exposure time) would complete the migration minimal loss of reserves.

By generalization, Eqs. (4–6) help us to provide biological meaning to the terms of the matrix **M**: any term of the form *dτ*${^*}$*/dm*_*j*_,* dτ*_*i*_*/dm*_*j*_ or *dτ*_*i*_*/dt*_*j*_ represents the effect of an environmental driver on the timing of a phenological event; hence, they are phenological components. Terms that contain the effect of an environmental variable on an instantaneous rate are eco-physiological components. By substitution we find that the terms of the matrix in Eq. (2) can be classified in two categories according to whether the component is eco-physiological (E) or phenological (P):

7 $$\left[\begin{array}{ccccc}E& 0& P& P& P\\ 0& E& P& P& P\\ 0& 0& P& P& 0\\ 0& 0& P& P& 0\\ 0& 0& 0& 0& P\end{array}\right]$$

### Case 2: multiple driver responses

Here we expand the previous case by looking at a response to the magnitude of two different drivers; i.e. keeping the levels of each driver constant over the duration of the experiment. Examples of this case are experiments quantifying the effect of temperature and nutrient load on body mass (e.g. in a rearing containers) or species richness (e.g. in mesocosms). This case is represented by the terms of first two rows of the matrix and the vectors of Eq. (2), with the terms of the remaining rows set to zero. Here, there are different scenarios, but we focus on the one highlighting the importance of biological time.

**Figure 4:**
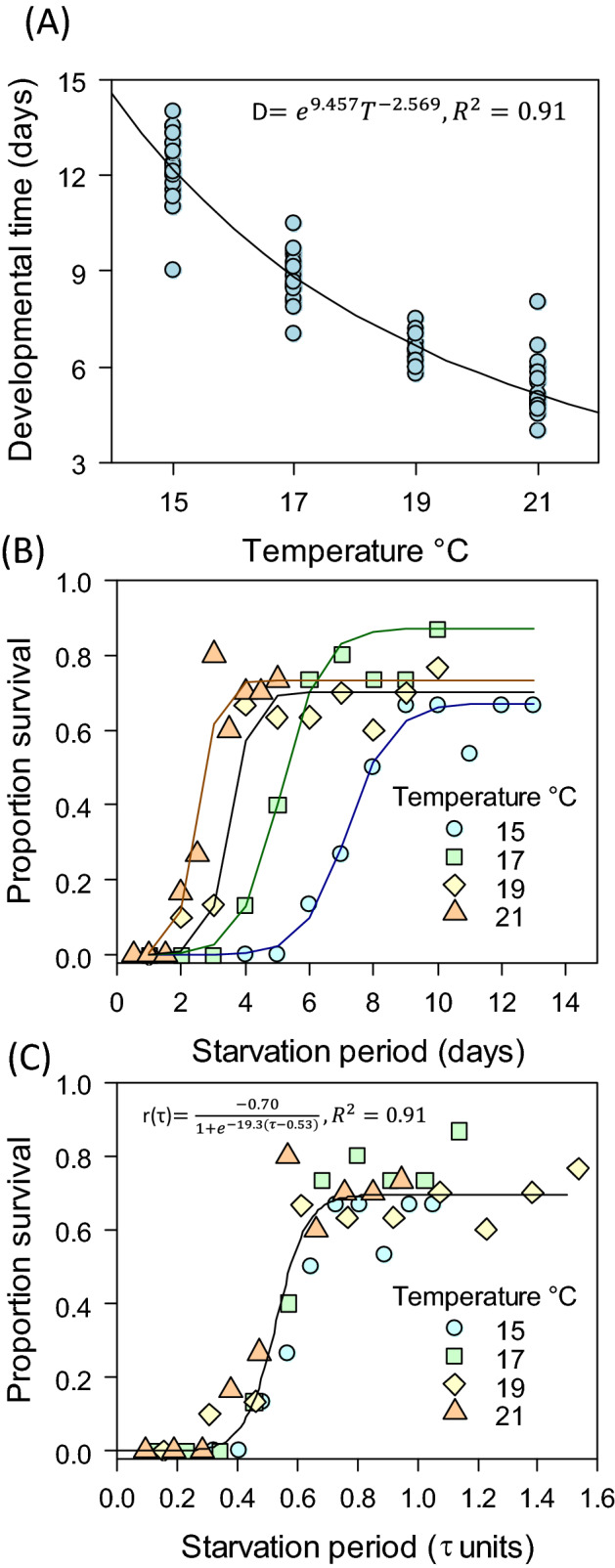
Case-3: Responses of food limitation. (**A**) Effect of temperature on the time needed by larvae to moult for developmental time, *D*(*T*)*.* (**B**) Proportion of survivors in response to temperature and the starvation period measured in clock time. Data were fitted with a Boltzmann sigmoidal function with parameters given in Supplementary Table 2. (**C**) Proportion of survivors vs time scale of starvation period in biological time. The equation obtained for the response in clock time (*t*), was *R *(*t,T*) = −0.70/(*1* + *f*), with *f* = 19.3[*t· e*^−9.457^* ·T*^2.569^)* − *0.53, *L* = *1/D(T)*.

Consider a case where biological time depends on the magnitude of the first driver while the response is explicitly driven by the magnitude of the second driver (Fig. 4). For instance, the response may be the survival rate of a host organism exposed to different temperature and parasitic load. The response in clock time is described as *R*(*m*_*P*_*, t*${^*}$)*.* The driver controlling the biological time is temperature (*m*_*T*_) while the parasitic load (*m*_*P*_) controls survival*.* In such case, d*τ*${^*}$*/*∂*m*_*P*_ = 0, *dR/dm*_*P*_* ≠ *0 and *dR/dm*_*T*_ = 0. Although by definition the response in clock time does not depend on *m*_*T*_* ,* it will do so in biological time. This is because, applying the matrix multiplication in Eq. (2), we obtain:

8a $$\frac{\partial R}{\partial {m}_{T}}=\frac{\partial r}{\partial {m}_{T}}+\frac{\partial r}{\partial \tau *}\cdot \frac{d\tau *}{d{m}_{T}}$$

8b $$0=\frac{\partial r}{\partial {m}_{T}}+\frac{\partial r}{\partial \tau *}\cdot \frac{d\tau *}{d{m}_{T}}$$

8c $$\frac{\partial r}{\partial {m}_{T}}=-\frac{\partial r}{\partial \tau *}\cdot \frac{d\tau *}{d{m}_{T}}$$

The second right-hand term in Eq. (8a) quantifies the effect of temperature on the response mediated by biological time. In order to better understand the responses, consider a simple linear response: *R* = *R*_*0*_* − m*_*P*_*·t*${^*}$ and notice that, for a fixed clock time (*t*${^*}$_*c*_) the effect of the magnitude of parasitism is constant (*dR/dm*_*P*_ = −*t*${^*}$_*c*_); hence, the response can be understood, geometrically, as a flat surface with slope not depending on temperature. Now, note that under the specific conditions of our example, *r* = *R*_*0*_* − m*_*P*_*·τ*${^*}$*/L*(*m*_*T*_)*.* Hence, for a fixed biological time (*τ*${^*}$_*c*_) we obtain ∂*r/*∂*m*_*P*_ = −*τ*${^*}$_*c*_*/L*(*m*_*T*_); i.e. the importance of the parasitic effect depends now on temperature. In addition, this example is valid for the case of additive effects of any two environmental drivers: assuming *R* = *R*_*0*_* − *(*a*_*1*_*·m*_*P*_ + *a*_*2*_*· m*_*T*_)*·t*${^*}$ *(a*_1_*, a*_2_ are constants*)*, we obtain *dR/dm*_*P*_ = −*a*_*1*_*t*${^*}$; however, ∂*r/*∂*m*_*P*_ = −*a*_*1*_*τ*${^*}$_*c*_*/L*(*m*_*T*_)*.* In words, additive effects observed in clock time become interactive in biological time. This is illustrated in the simulation (Supplementary code 2) depicted in Fig. 4: the response in clock time depends on a single driver (parasite load); however, the response in biological time is interactive, i.e. the effect of parasite load depends on temperature.

### Case 3: role of clock and biological time scale of fluctuation

Previous examples did not consider, the time scale of the fluctuations as drivers of the response. Here we explore how a biological variable (= survival rate) responds to different levels of magnitude of a driver (= temperature) and to simultaneously changing the time scale of a fluctuation (from clock to biological time) of a second driver (= food limitation). As model, we use larval stages of a crab because there is sufficient information on the effect of temperature and food levels on survival and the timing of moulting^33,34^.

We performed the so-called point-of-reserve-saturation experiment (PRS^35^), i.e. exposing groups of recently hatched larvae of the crab *Hemigrapsus sanguineus* to different initial feeding periods (= our time scale of fluctuation), after which they were starved until they either died or moulted to the second larval stage (Supplementary Fig. 1). *H. sanguineus* is originated from East Asia but has invaded the Atlantic shores of North America and North Europe^36,37^. This experiment was carried out at 4 temperature levels (15–21 °C), within the range of thermal tolerance of larvae of this species, i.e. where the magnitude of temperature does not affect survival^38,39^. In addition, because there is a single level of food limitation (= starvation), the magnitude of food limitation (m_*F*_) is not considered as a variable in the example.

The response variable was the proportion of first stage larvae surviving the moulting event to the second stage, set to biological time *τ*${^*}$ = 1. In response to different starvation periods (preceded by feeding), the survival shows a sigmoidal pattern^35^, characterised by a parameter, PRS_50_. This is the point of development where larval reserves are “saturated”; i.e. enough reserves have been accumulated during the previous feeding period to ensure survival and moulting to the next stage.

Under the conditions of the experiment, the survival proportion (= R) is driven only by the time scale of a fluctuation (here *t*_1_ = *t, τ*_*1*_ = *τ* for simplicity), characterised by the starvation period; hence, *R* = *R*(*t*) = *r*[*τ*(*t*)] given that there is a single time of observation fixed to *τ*${^*}$ = 1. Because biological time does not depend *t*, we get *L* = d*τ/*d*t* and:

9 $$\frac{dR}{dt}=\frac{\partial r}{\partial \tau }\cdot \mathcal{L}({m}_{2})$$

Equation (9) is represented in the PDE by the terms of row 3 and column 4 of **M** multiplied by the term of row 3 of the column vector **r**; *dτ/dt* = *L*(*m*),* m* represents the magnitude of temperature.

The relationship between biological time and temperature was best explained by a power function *D(T)* = *aT*^*b*^ (Fig. 4A, Supplementary Table 1, Supplementary Fig. 2), in consistence with previous studies^36,40^. The interaction between starvation time and temperature was weak (Supplementary Fig. 3); best models retained starvation time only at 21 °C where the percentage of explained variance was still low (R^2^ < 0.2). The full range of starvation times resulted in a variation of developmental time of < 2 days, while the full range of temperature used resulted in variations of 8 days (range 5–14 days); hence, we approximated the model as *L* depending on temperature as *L* = *1/*(*aT*^*b*^).

Survival showed an S-shape pattern consistent with results found for other species^35^. When the starvation time was expressed in clock time (*PRS*_*50*_ = *t*_*50*_ in days*)* there was a dilation/contraction effect of the response curve, quantified by the *PRS*_*50*_ and driven by the effect of temperature on biological time (Fig. 4B, Supplementary Table 2). When time was expressed in biological time units (*PRS*_*50*_ = *τ*_*50*_), a single response curve explained 91% of total variation (Fig. 4C, Supplementary Table 3), irrespective of temperature. The estimate of parameters by temperature showed PRS_50_ in the range of 0.47–0.58% of moulting time with a slight decrease towards higher temperatures (Supplementary Table 4); the range of percent values found here is also consistent with findings in other species (40–60%)^35^. There were therefore important differences in the effect of temperature on estimates of PRS_50_ depending on the choice between time scale (Supplementary Table 5). In synthesis, in the biological time scale we found a simple function showing that the PRS_50_ was less responsive to a change in temperature than in clock time; we will address this point in the discussion in the context of physiological time keeping mechanisms.

### Case 4: biological time depends on the time scale of the fluctuation

Here, we generalise the above cases by considering situations where both the magnitude and time scale of an environmental fluctuation drive biological time. For simplicity, we consider a single driver. In such case, *L* = *L*(*m,t*) and the PDEs reduce to:

10a $$\frac{\partial R}{\partial t}=\frac{\partial r}{\partial \tau }\cdot \mathcal{L}+\frac{\partial r}{\partial \tau }\cdot t\cdot \frac{d\mathcal{L}}{dt}$$

10b $$\frac{\partial R}{\partial m}=\frac{\partial r}{\partial m}+\frac{\partial r}{\partial \tau }\cdot t\cdot \frac{d\mathcal{L}}{dm}+\frac{\partial r}{\partial \tau *}\cdot {t}^{^*}\cdot \frac{d\mathcal{L}}{dm}$$

We now consider a response interpreted as a decay in performance of an organism, where longer time scales of the fluctuation increase the biological time, as expected for cases where organisms are exposed to suboptimal conditions (e.g. food limitation experienced over a given time scale). There are obviously many possible scenarios but for better understanding, we consider Cases 4A-D, where *dL/dt* is negative, reducing performance and where the functions linking *m* and *t* with *L* act additively or multiplicatively. Additive responses are will be illustrated with *L* = [*k*_*1*_*/m* + *k*_*2*_*/t*]*,* while multiplicative responses will be illustrated *L* = *k*_*3*_*/mt,* with *k*_*1*_,* k*_*2*_ and *k*_*3*_ as constants.

The first two cases focus on Eq. (10a), which may be considered as an extension of Case 3. Case 4A: additive response: in such case *dL/dt* depends only on *t* and we get that *dR/dt* and *dr/dτ* differ by a factor *k*_*1*_*/m*:

$$\frac{\partial R}{\partial t}=\frac{\partial r}{\partial \tau }\cdot \frac{{k}_{1}}{m}$$

In addition, if *L* only depends on *t*, the result is that the response in clock time does not depend on the time scale of fluctuation (*dR/dt* = 0) because the terms of Eq. (10a) associated to L cancel out*.* However*,* the response in biological time does not need to be zero (*dr/dτ* may not be zero)*.* For instance, assume that *r(m, τ)* = *exp(−ω·m·τ)*, and *L* = *k*_*2*_*/t* , with *ω* = constant. We obtain *R* = *exp(−ω·m·k*_*2*_*)* and *dR/dt* = *0,* while *dr/dτ* = *−ω·m·exp(−ω·m·τ).*

Case 4B: Multiplicative response of *m* and *t* in *L.* In such case, *dR/dt* = *0* because the terms associated to *L* cancel out, while *dr/dτ* may not be zero.

The next two cases focus on Eq. (10b), which is an extension of cases 1 and 2. The effect of the time scale of the fluctuation depends again on how it relates, through *L*, to the effect of the magnitude of the fluctuation. We interpret the response as a decay in performance (or fitness), contributed by the “ecophysiological” and “phenological” components:

11 $$\frac{dR}{dm}=\frac{\partial r}{\partial \mu }\cdot \frac{d\mu }{dm}+\left\{\frac{\partial r}{\partial \tau }\cdot t\cdot \frac{\partial \mathcal{L}}{\partial m}+\frac{\partial r}{\partial {\tau }^{^*}}\cdot {t}^{^*}\cdot \frac{\partial \mathcal{L}}{\partial m}\right\}$$

In Eq. (11), the phenological component (within the brackets) is driven by two terms. The expression *t·dL/dm* shows that the time scale of the fluctuation act as increasing the exposure to the suboptimal conditions and contributes to a further reduction in performance. Equation (11) takes different forms depending on whether the functions linking *t* and *m* with *L* are additive or multiplicative.

Case 4C: when the response is additive, the phenological contribution is proportional to the time scale of the fluctuation which then contributes to a further reduction of the performance:

$$\frac{dR}{dm}=\frac{\partial r}{\partial \mu }\cdot \frac{d\mu }{dm}-\frac{{k}_{1}}{{m}^{2}}\left\{\frac{\partial r}{\partial \tau } t+\frac{\partial r}{\partial {\tau }^{^*}}{t}^{^*}\right\}$$

Case 4D. When the response is multiplicative, the phenological component responds non-linearly to the time scale of the fluctuation.

$$\frac{dR}{dm}=\frac{\partial r}{\partial \mu }\cdot \frac{d\mu }{dm}-\frac{{k}_{3}}{{m}^{2}}\left\{\frac{\partial r}{\partial \tau }+\frac{\partial r}{\partial {\tau }^{^*}}\cdot \frac{{t}^{^*}}{t}\right\}$$

In both 4C and 4D the effect of the magnitude, *m*, on the response depends on whether the fluctuation is quantified in terms of clock or biological time. For example, when *L* = *k*_*1*_*/m or L* = *k*_*3*_*/mt, dR/dm* does not depend on* t* while *dr/dm* depends on *τ.* Overall, Cases 4A-D further extend the relevance of cases 1 and 2 in understanding the effects of fluctuating environmental drivers on the responses.

## Discussion and conclusions

We have introduced a mathematical framework to better understand and quantify the responses of biological systems to fluctuations in climate driven stressors. Central to that framework is the need to consider biological time as playing a role in driving ecological and evolutionary processes^20^. We did not consider the average magnitude of the fluctuation because responses to fluctuations can differ from responses to the average in two different ways. First, under extreme fluctuations critical tolerance levels may be surpassed leading to the collapse or irreversible shift of the biological system under study. The most obvious case is when the temperature surpasses critical tolerance levels leading to death^42^ but experiencing less extreme levels may lead to negative carry-over effects or acclimatory responses^15^, not present when average conditions are experienced. Second, when the relationships between environmental drivers (e.g. temperature) and biological responses (e.g. physiological performance) is non-linear, average conditions do not predict well the expected response to a fluctuating environment^13,17^.

We started with the simple Case 1 of a single variable and no fluctuation. This case may be considered as trivial but helped as a step to understand more complex cases. For instance, through Case 1 we noted that when the matrix **M** of the PDE is not the identity matrix, simultaneous events in biological time are not so in clock time; this known observation in experimental research was then identified geometrically as the difference between a curve and straight lines when plotting the response in the space defined the magnitude of an environmental variable and the time scale of observation (Fig. 2). With further analysis of Case 1, we noted that the **M** contained two types of terms interpreted as eco-physiological and phenological effects. The specific example of the migrating organism provided further biological interpretation to the components of the differential equation. We focused on the negative effects of temperature (biomass loss) and then noted that adaptations should minimise either one or both the phenological and the physiological components if biomass loss were to be minimised. This is for instance the case of the evolution of early life histories of marine invertebrates to habitats characterised by limited food availability^43^. Where food is available, marine invertebrate tend to develop through feeding larval stages; however, where food is too limiting, most species develop through non-feeding larvae with abbreviated larval phase. In such case, the allocation of maternal reserves into eggs contributes to minimise both the eco-physiological a phenological components with respect to survival, because both the mortality rates and developmental time are independent of food availability.

In Case 2, we introduced the magnitude of a second variable, to explore more complex scenarios studied through factorial experiments carried out under constant conditions; i.e. not yet considering the time scale of a fluctuation. Here, the presence of an interactive response depended on the scale used to measure time. This finding is central to climate change biology, given the interest on interactive effects of multiple environmental variables on biological systems^12,18,19,44^. A critical question is whether effects are additive or whether they are antagonistic or synergistic. Additive effects refer to situation where the response can be modelled from the isolated effect of each single variable; many biological responses are however synergistic or antagonisitic^44^. Synergistic responses occur when the combined effects are larger than the expected contribution of each separate variable. Synergistic responses are critical when they are negative, for instance the combined effect of habitat loss and an environmental stressor, as they can drive ecosystem collapse. By contrast, antagonistic responses imply a mitigation effect. For management, it is essential to get the response right because resources for actions are limited and disrupting synergies may be considered a priority^51^. In such context our findings suggest that management depends on using the correct time frame to measure the response. Interactive effects also change when responses at low levels of organization (e.g. consumer resource functional responses) are used to predict those at higher levels (e.g. population dynamics^45^), because of the non-linear nature of the function mapping the response across levels. In our case there is a non-linearity in that the components of **M** are partial derivatives which depend on the predictors (i.e. the original functions are non-linear).

We used Case 3 to explore responses including the time scale of fluctuation and to better understand how experimental results are interpreted in the light of the PDE. We studied responses in larval stages because of the relevance of marine larvae in driving climate-change effects on marine organisms: most marine organisms (e.g. mussels, crabs, fish) develop through a pelagic larval stage, and larval dynamics affect species range^38,39^, population dynamics^46^, connectivity^47^ and community structure^48^. Warming provides a new context where larvae need to cope with fluctuations in e.g. food abundance (or other variables) in a scenario of increased metabolic demands due to higher temperatures^49^.

Case 3 highlighted the importance of understanding how temperature drives time keeping mechanisms in biological systems, understood as those responsible for setting the pace and regulating the timing of life history events^50^. For Case 3, the phenological component of the PDEs captured the effect of temperature on *PRS*_*50*_ which instead reflects hormonal control of the so called “D_0_-threshold”. This threshold is surpassed when moulting hormones are triggered and the premoult period starts^51^; after *D*_*0*_, development proceeds at a rate that is independent of food levels and larvae will moult. Case 3 therefore highlights the importance of understanding how temperature drives the hormonal regulation of moulting, for the formulation of mechanistic models predicting survival. By extension, knowledge of role of hormones and other signalling mechanisms^50,52,53^ should help the formulation of models in other species. In cases where organisms undergo acclimation, an important question is how the time scale of acclimation relates to time keeping mechanisms, including hormonal processes and metabolic rates^31^. Acclimation speed correlates negatively with body size, most likely driven by the positive effect of metabolic rates on acclimation speed^54^. Perhaps acclimation time, as a fraction of developmental time, varies little with temperature or alternatively, acclimation time and the developmental processes setting phenological events have different sensitivities to temperature.

In Case 4, we introduced effects of the time scale of a fluctuation on the function *L* and found equations that may be considered extensions of the previous cases. A critical question is to determine situations when responses may follow Cases 1–3, 4 or be further simplified into the trivial case. An important point is therefore to determine how and when developmental time depends on the time scale of the fluctuation being experienced. For instance, we find that dependencies on degree days^41^ fit within Cases 1–3 (Supplementary note 2, Supplementary Fig. 4). However, whether the developmental time is driven by time scale of the fluctuation depends on the timing of the fluctuation in relation to size thresholds reached as organisms grow^55–57^. Our work suggest that we need to understand how such thresholds relate to time-keeping mechanisms.

We used the PDEs to understand the importance of the choice of time scale, within an experimental setting. In addition, Case 3 suggest the set of conditions where simple models predict responses to environmental fluctuations. In the example, the response may be approximated by a function quantifying the relationship between temperature and biological time and a second function controlling the timing of the starvation period. Notice that under the range of temperatures considered, the transformation from clock to biological time led to a simple model with high predicting capacity (> 90% of explained variation) although it ignores the significant (but small) effects of food limitation on developmental time (Supplementary Fig. 3). In similar cases, data of the response from a narrow temperature range would give an approximation of ∂*r/*∂*τ* when scaled in biological time. In that case, additional data on the effect of temperature on biological time may be used predict the response. An important point is the set of conditions where equation-8 may be used with safety. For our experimental system, we hypothesise that equation-2 had a good fit because the developmental time varied little with the time of starvation and the range of temperatures was within the so called “pejus range”^58^ i.e. where survival was high irrespective of the magnitude of temperature (*dR/d*m_T_ ≈0). However, assumptions are not valid if the response fall within Case 4 or at temperatures beyond the pejus threshold where a change in temperature have strong effects on the response.

Under the conditions of equation-8, one may combine mechanistic sub-models as modules (e.g. for each of the partial derivatives). In Case 3, the first module is given by *L* which may be modelled from metabolic theories (MTE^32,33^). For instance, in the MTE, the effect of temperature on biological time, is represented by the Arrhenius equation, which instead will determine *L*. For the second module (represented by ∂*r/*∂*τ*)*,* we can associate the response to hormonal control of development which drive the timing the switch of the sigmoid survival function. More in general, sigmoid responses are characteristic of populations or ecosystems exhibiting regime or phase shifts^59,60^. At the population level, phase shifts reflect an unstable equilibrium (saddle points) point driven by thresholds associated to density-dependent changes in mating and reproduction. Hence, the mechanisms associated to such phase shifts would be captured in the response function expressed on biological time scale (e.g. the generation time for population level responses).

There are two important points concerning relevance of our approach to characterizing biological responses to environmental fluctuations in the field. First, our main finding, i.e. that responses to environmental fluctuations depends on the scale used to measure time, is valid for both field and experimental conditions. The use of the experimental set up only facilitates teasing apart the independent contributions of each of the predictors (i.e. the magnitudes and time scales of fluctuations). However, whether one can directly apply the equations straight away to field conditions, depends on meeting the assumptions used to formulate the equations; in this sense our approach is not different from any other experimental approach and the usual recommendations apply^18^. There are three assumptions: (1) the right few predictors are identified (e.g. magnitudes and times scales of temperature and any other factor). (2) No covariation among predictors; (3) fluctuations characterised by well-defined values of predictors. A first challenge in field applications is the complexity shown by natural environmental fluctuations. For instance, real fluctuations (e.g. heatwaves) consist of a sequence of oscillations; in addition, fluctuations may be characterised by descriptors other than the period and amplitude (e.g. the rate of daily temperature increase in tropical habitats)^20^. A second challenge is that environmental variables covary in the field^12^. This include situations, not considered here, where different environmental variables fluctuate sequentially^12,31^, and an additional time scale must be included in the PDEs (i.e. the one separating the fluctuations). Third, in the field, fluctuations may be characterised by means and variances of the predictor values and attempts to model the average biological response need to consider issues associated to non-linear responses^13,17^. However, the most likely scenario is that field observations inform the design of future experiments. For example, field studies can identify the main environmental variables, the most important traits characterising the fluctuations, whether fluctuations of different variables occur sequentially or simultaneously^12,18^.

Another important question is what time frame should we choose. The selection of the appropriate reference frame will depend on the question asked by the researcher. In experiments aimed at determining the effects of environmental fluctuations on body size or survival at e.g. maturation, biological time will be the obvious choice. Biological time will be a choice in situations where organisms experience habitat shifts through the life cycle, for example in species where the larval habitat is aquatic and the adult habitat is terrestrial. In such case, once the aquatic larvae metamorphose to a terrestrial juvenile stage, the importance of the larval habitat conditions for the survival of the juvenile is likely to be low; hence, what matters are the conditions experienced as larvae up to the time of metamorphosis. There are however cases where the decision is less clear. For example, where larvae and adults coexist or where key environmental variables (e.g. weather conditions) can affect both the larval and adult habitat. Another example is the one given by, mesocosm experiments, used to study the effect of warming on populations and communities^18^; under warming, it is likely that populations fluctuate over more generations that in the absence of warming. If the number of generations is important, it will be helpful to analyse the responses considering both clock and biological time (i.e. after a fixed number of generations). The same experiment may be designed to understand the importance of a fluctuation occurring at a characteristic clock time scale, associated to e.g. seasonal fluctuations or 5-day long heatwaves; in such case, the scale of the fluctuation should be kept in clock time. Overall, we will profit from analysing the response in both time frames, as they will provide different pieces of information.

## Methods

Experiments were carried out in automated fully programmable incubators (RUMED-EcoLine^R^). At each combination of temperature and starvation period, three replicate groups (10 larvae each) were kept in well oxygenated and filtered natural seawater (mesh = 1 μm, salinity = 32.5 PSU) in glass vials of 100 ml. Water and food were changed every day (except at 21 °C where food was checked every ~ 12 h, at 7:00 and 18:00 h); at such times larvae were checked for moulting or mortality (dead larvae were removed from cultures). Larvae were fed freshly hatched *Artemia* sp nauplii, provided *at libitum* (density 5 nauplii per ml) during the feeding periods.

The effect of temperature (T) or feeding period on developmental time (D) were evaluated through model selection^61,62^ using general least squares for model fitting and Akaike information criterium (AIC) for model comparison. Analyses were carried out in R using the package nlme^61^. Four models were compared, i.e. linear ($D=-a \cdot T$+b), exponential ($D=a\cdot {e}^{-b\cdot T}$), power ($D=a\cdot {T}^{-b}$) and Arrhenius ($D=a\cdot {e}^{\frac{b}{(T+273}}$), where *a* and *b* are constants. Models were fitted after appropriate transformations, log(D) for exponential, log(D)-log(T) for power and log(D) vs 1/(T + 273) for Arrhenius model.

Effects of initial feeding periods on survival were evaluated using the sigmoidal dose response function:

$$f(x)={f}_{m}+ \frac{{f}_{M}-{f}_{m}}{1+{e}^{-(x-{PRS}_{50})/k}}$$

where *f*_*m*_ and *f*_*M*_ are the asymptotic minima and maxima respectively, k is the slope parameter and PRS_50_ is the timing of the inflection point where *f(PRS*_*50*_*)* = *f(x*_*M*_*)/2*. Model fitting was carried out by non-linear regression (in GraphPad Prism software), with feeding period expressed in both chronological and biological time scales. When the biological time scale was used, a single model was fitted to data from all temperatures. In that case, there were sufficient degrees of freedom to enable appropriate estimation of the four model parameters. In addition, separate models were fitted by temperature using at biological and chronological time. For those models, the estimation of some parameters was unreliable (e.g. extremely large confidence intervals); hence, we focused on estimating PRS_50_, which is the parameter determining the time of the inflexion point in the curve. Therefore, we set *f*_*m*_ *= *0 and *f*_*M*_ = *M*, with *M* being the average survival of the last three points. In addition, for 19 and 21 °C we set *k* to a constant value obtained after our initial attempt to estimate k.

## Supplementary Information

Supplementary Information.

## Data Availability

Data will be available in the portal PANGEA.

## References

[1] Wigley TM (2005). The climate change commitment. Science.

[2] Chen PY, Chen CC, Chu L, Mc CB (2015). Evaluating the economic damage of climate change on global coral reefs. Glob. Environ. Change.

[3] UN. *2015 Transforming our World: the 2030 Agenda for Sustainable Development*.

[4] Baselga D, Araujo M (2009). Individualistic vs community modelling of species distributions under climate change. Ecography.

[5] Burrows MT (2011). The pace of shifting climate in marine and terrestrial ecosystems. Science.

[6] Moritz C, Agudo R (2013). The future of species under climate change: Resilience or decline?. Science.

[7] Bennett S, Wernberg T, ArackalJoy B, de Bettignies T, Campbell AH (2015). Central and rear-edge populations can be equally vulnerable to warming. Nat. Commun..

[8] Levins R (1968). Evolution in Changing Environments.

[9] Sæther B-E, Engen S (2015). The concept of fitness in fluctuating environments. Trends Ecol. Evol..

[10] Bernhardt JR, O'Connor MI, Sunday JM, Gonzalez A (2020). Life in fluctuating environments. Philos. Trans. R. Soc. B.

[11] Burton T, Lakka H-K, Einum S (2020). Measuring phenotypes in fluctuating environments. Funct. Ecol..

[12] Gunderson A, Armstrong E, Stillman J (2016). Multiple stressors in a changing world: the need for an improved perspective on physiological responses to the dynamic marine environment. Annu. Rev. Mar. Sci..

[13] Kroeker KJ (2020). Ecological change in dynamic environments: Accounting for temporal environmental variability in studies of ocean change biology. Global Change Biol..

[14] Britton D, Cornwall CE, Revill AT, Hurd CL, Johnson CR (2016). Ocean acidification reverses the positive effects of seawater pH fluctuations on growth and photosynthesis of the habitat-forming kelp, *Ecklonia radiata*. Sci. Rep..

[15] Jarrold MD, Humphrey C, McCormick MI, Munday PL (2017). Diel CO_2_ cycles reduce severity of behavioural abnormalities in coral reef fish under ocean acidification. Sci. Rep..

[16] Thompson RM, Beardall J, Beringer J, Grace M, Sardina P (2013). Means and extremes: Building variability into community-level climate change experiments. Ecol. Lett..

[17] Denny M (2019). Performance in a variable world: Using Jensen’s inequality to scale up from individuals to populations. Conserv. Physiol..

[18] Boyd PW (2018). Experimental strategies to assess the biological ramifications of multiple drivers of global ocean change—A review. Global Change Biol..

[19] Orr JA (2020). Towards a unified study of multiple stressors: divisions and common goals across research disciplines. Proc. R. Soc. B.

[20] Hobday AJ (2016). A hierarchical approach to defining marine heatwaves. Prog. Oceanogr..

[21] Post, E. Time in ecology: A theoretical framework. in *Monographs in Population Biology*. Vol. 61. (Princeton University Press, 2020).

[22] Stearns S (1986). Evolution of Life Histories.

[23] Roff D (1992). Evolution of Life Histories. Theory and analysis.

[24] Caswell H (2001). Matrix Population Models.

[25] Silby R, Brown J, Kodrik-Brown A (2012). Metabolic Ecology. A Scaling Approach.

[26] Carroll SP, Hendry AP, Reznick DN, Fox CW (2007). Evolution on ecological time-scales. Funct. Ecol..

[27] Chevin L-M, Lande R, Mace GM (2010). Adaptation, plasticity, and extinction in a changing environment: Towards a predictive theory. PLoS Biol.

[28] Gerken AR, Eller OC, Hahn DA, Morgan TJ (2015). Constraints, independence, and evolution of thermal plasticity: Probing genetic architecture of long- and short-term thermal acclimation. Proc. Natl. Acad. Sci..

[29] Hoffmann AA, Sgro CM (2011). Climate change and evolutionary adaptation. Nature.

[30] Donelson JM, Salinas S, Munday PL, Shama L (2018). Transgenerational plasticity and climate change experiments: Where do we go from here?. Global Change Biol..

[31] Jackson MC, Pawar S, Woodward G (2021). Temporal dynamics of multiple stressor effects: from individuals to ecosystems. Trends Ecol. Evol..

[32] Brown JH, Gillooly JF, Allen AP, Savage M, West GB (2004). Towards a metabolic theory of ecology. Ecology.

[33] O'Connor M (2007). Temperature control of larval dispersal and the implications for marine ecology, evolution, and conservation. Proc. Natl. Acad. Sci..

[34] Anger, K. The biology of decapod crustacean larvae. in *Crustacean Issues 14*. (Balkema, 2001).

[35] Anger K (1987). The D_0_ threshold: a critical point in the larval development of decapod crustaceans. J. Exp. Mar. Biol. Ecol..

[36] Epifanio C (2013). Invasion biology of the Asian shore crab *Hemigrapsus sanguineus*: A review. J. Exp. Mar. Biol. Ecol..

[37] Geburzi J, Brandis D, Buschbaum C (2018). Recruitment patterns, low cannibalism and reduced interspecific predation contribute to high invasion success of two Pacific crabs in northwestern Europe. Estuar. Coast. Shelf Sci..

[38] Epifanio, C., Dittel, A., Park, S., Schwalm, S. & Fouts, A. Early life history of *Hemigrapsus sanguineus*, a non-indigenous crab in the Middle Atlantic Bight (USA). *Mar. Ecol. Prog. Ser*. **170**, 231–238 (1998).

[39] Giménez L (2020). Exploring larval phenology as predictor for range expansion in an invasive species. Ecography.

[40] Dawirs R (1985). Temperature and larval development of *Carcinus maenas* (Decapoda) in the laboratory; predictions of larval dynamics in the sea. Mar. Ecol. Prog. Ser..

[41] Bonhomme R (2000). Bases and limits to using “degree.day” units. Eur. J. Agron..

[42] Lynch HJ (2014). How climate extremes-not means-define a species’ geographic range boundary via demograhic tiooing point. Ecol. Monogr..

[43] Anger K (1995). The conquest of freshwater and land by marine crabs: adaptations in life-history patterns and larval bioenergetics. J. Exp. Mar. Biol. Ecol..

[44] Côté IM, Darling ES, Brown CJ (2016). Interactions among ecosystem stressors and their importance in conservation. Proc. R. Soc. B.

[45] De Laender F (2018). Community- and ecosystem-level effects of multiple environmental change drivers: Beyond null model testing. Glob. Change Biol..

[46] Roughgarden J, Gaines S, Possingham H (1988). Recruitment dynamics in complex life cycles. Science.

[47] Cowen RK, Paris CB, Srinivasan A (2006). Scaling of connectivity in marine populations. Science.

[48] Connolly SR, Roughgarden J (1999). Theory of marine communities: Competition, predation, and recruitment-dependent interaction strength. Ecol. Monogr..

[49] Torres G, Giménez L (2020). Temperature modulates compensatory responses to food limitation at metamorphosis in a marine invertebrate. Funct. Ecol..

[50] Helm B (2013). Annual rhythms underlie phenology: biological time-keeping meets environmental change. Proc. R Soc. B.

[51] Anger K, Spindler K-D (1987). Energetics, moult cycle and ecdysteroid titers in spider crab (*Hyas araneus*) larvae starved after the D_0_ threshold. Mar. Biol..

[52] Crespi EJ, Williams TD, Jessop TS, Delehanty B (2013). Life history and the ecology of stress: How do glucocorticoid hormones influence life-history variation in animals?. Funct. Ecol..

[53] Lema SC (2014). Hormones and phenotypic plasticity in an ecological context: Linking physiological mechanisms to evolutionary processes. Integr. Comp. Biol..

[54] Rohr JR (2018). The complex drivers of thermal acclimation and breath in ectotherms. Ecol. Lett..

[55] Hentschel BT (1999). Complex life cycles in a variable environment: Predicting when the timing of metamorphosis shifts from resource dependent to developmentally fixed. Am. Nat..

[56] Day T, Rowe L (2002). Developmental thresholds and the evolution of reaction norms for age and size at life-history transitions. Am. Nat..

[57] Nilsson-Örtman V, Rowe L (2021). The evolution of developmental thresholds and reaction norms for age and size at maturity. Proc. Natl. Acad. Sci..

[58] Pörtner H-O, Knust R (2007). Climate change affects marine fishes through the oxygen limitation of thermal tolerance. Science.

[59] May RM (1977). Thresholds and breakpoints in ecosystems with a multiplicity of stable states. Nature.

[60] Scheffer M (2021). Critical Transitions in Nature and Society.

[61] Pinheiro, J., Bates, D., DebRoy, S. & Sarkar, D. *R Core Team. nlme: Linear and Nonlinear Mixed Effects Models*. *R Package Version 3.1–137* (2018).

[62] Zuur A, Ieno E, Walker N, Savaliev A, Smith G (2009). Mixed Effect Models and Extensions in Ecology with R.

