# Supplementary material for: A framework to understand the role of biological time in responses to fluctuating climate drivers
Source: Sci Rep. 2022 Jun 21;12:10429. doi: 10.1038/s41598-022-13603-5 (PMC9213464; doi:10.1038/s41598-022-13603-5)
Supplement: Supplementary file 1 — Supplementary Information. [file 41598_2022_13603_MOESM1_ESM.docx]

**A framework to understand the role of biological time in responses to fluctuating climate drivers**

**Supplementary materials**

1. **Supplementary note 1: Full derivation of PDEs**

The response is considered a function of the time of observation (*t*, τ**), the magnitudes (*m_1_, m_2_*) and time scales (*t_1_, t_2_, τ_1_, τ_2_*) of fluctuation of two environmental drivers: *R(t*, t_1_, t_2_, m_1_, m_2_) = r[τ*(t*), τ_1_(t_1_), τ_2_(t_2_), m_1_, m_2_]*. The times are expressed in clock (t’s) and biological time(*τ’s*). Differentiating, we obtain:

$$\frac{dR}{dm_{1}}=\frac{\partial r}{\partial m_{1}}+\frac{\partial r}{\partial m_{2}}\cdot\frac{dm_{2}}{dm_{1}}+\frac{\partial r}{\partial\tau_{1}}\cdot\frac{d\tau_{1}}{dm_{1}}+\frac{\partial r}{\partial\tau_{2}}\cdot\frac{d\tau_{2}}{dm_{1}}+\frac{\partial r}{\partial\tau^{*}}\cdot\frac{d\tau^{*}}{dm_{1}}$$

$$\frac{dR}{dm_{2}}=\frac{\partial r}{\partial m_{2}}+\frac{\partial r}{\partial m_{1}}\cdot\frac{dm_{1}}{dm_{2}}+\frac{\partial r}{\partial\tau_{1}}\cdot\frac{d\tau_{1}}{dm_{2}}+\frac{\partial r}{\partial\tau_{2}}\cdot\frac{d\tau_{2}}{dm_{2}}+\frac{\partial r}{\partial\tau^{*}}\cdot\frac{d\tau^{*}}{dm_{2}}$$

$$\frac{dR}{dt_{1}}=\frac{\partial r}{\partial m_{1}}\frac{dm_{1}}{dt_{1}}+\frac{\partial r}{\partial m_{2}}\cdot\frac{dm_{2}}{dt_{1}}+\frac{\partial r}{\partial\tau_{1}}\cdot\frac{d\tau_{1}}{dt_{1}}+\frac{\partial r}{\partial\tau_{2}}\cdot\frac{d\tau_{2}}{dt_{1}}+[\frac{\boldsymbol{\partial r}}{\boldsymbol{\partial}\boldsymbol{\tau}^{\boldsymbol{*}}}\boldsymbol{\cdot}\frac{\boldsymbol{d}\boldsymbol{\tau}^{\boldsymbol{*}}}{\boldsymbol{d}\boldsymbol{t}_{\boldsymbol{1}}}\boldsymbol{]}$$

$$\frac{dR}{dt_{2}}=\frac{\partial r}{\partial m_{1}}\frac{dm_{1}}{dt_{2}}+\frac{\partial r}{\partial m_{2}}\cdot\frac{dm_{2}}{dt_{2}}+\frac{\partial r}{\partial\tau_{1}}\cdot\frac{d\tau_{1}}{dt_{2}}+\frac{\partial r}{\partial\tau_{2}}\cdot\frac{d\tau_{2}}{dt_{2}}+[\frac{\boldsymbol{\partial r}}{\boldsymbol{\partial}\boldsymbol{\tau}^{\boldsymbol{*}}}\boldsymbol{\cdot}\frac{\boldsymbol{d}\boldsymbol{\tau}^{\boldsymbol{*}}}{\boldsymbol{d}\boldsymbol{t}_{\boldsymbol{2}}}\boldsymbol{]}$$

$$\frac{dR}{dt^{*}}=\{\frac{\boldsymbol{\partial r}}{\boldsymbol{\partial}\boldsymbol{m}_{\boldsymbol{1}}}\frac{\boldsymbol{d}\boldsymbol{m}_{\boldsymbol{1}}}{\boldsymbol{d}\boldsymbol{t}^{\boldsymbol{*}}}\boldsymbol{+}\frac{\boldsymbol{\partial r}}{\boldsymbol{\partial}\boldsymbol{m}_{\boldsymbol{2}}}\boldsymbol{\cdot}\frac{\boldsymbol{d}\boldsymbol{m}_{\boldsymbol{2}}}{\boldsymbol{d}\boldsymbol{t}^{\boldsymbol{*}}}\boldsymbol{\}+[}\frac{\boldsymbol{\partial r}}{\boldsymbol{\partial}\boldsymbol{\tau}_{\boldsymbol{1}}}\boldsymbol{\cdot}\frac{\boldsymbol{d}\boldsymbol{\tau}_{\boldsymbol{1}}}{\boldsymbol{d}\boldsymbol{t}^{\boldsymbol{*}}}\boldsymbol{+}\frac{\boldsymbol{\partial r}}{\boldsymbol{\partial}\boldsymbol{\tau}_{\boldsymbol{2}}}\boldsymbol{\cdot}\frac{\boldsymbol{d}\boldsymbol{\tau}_{\boldsymbol{2}}}{\boldsymbol{d}\boldsymbol{t}^{\boldsymbol{*}}}]+\frac{\partial r}{\partial\tau^{*}}\cdot\frac{d\tau^{*}}{dt^{*}}$$

The terms in square brackets are zero because of the mathematical definition of *τ_1_, τ_2_* and *τ**:

$$\frac{d\tau^{*}}{dt_{1}}=0,\frac{d\tau^{*}}{dt_{2}}=0,\frac{d\tau_{1}}{dt^{*}}=0, \frac{d\tau_{2}}{dt^{*}}=0,$$

Neither *τ_1_* nor *τ_2_* depend on t* because the latter refers to a time after the fluctuations occurred. Likewise, because *τ** corresponds to a time after the fluctuations occurred it does not depend on *t_1_* or *t_2_*_._

The terms in braces, {}, are zero because the magnitudes *m_1_* and *m_2_* are defined as associated to *t_1_* and *t_2_* and hence do not vary with *t**. Therefore:

$$\frac{dm_{1}}{dt^{*}}=0, \frac{dm_{2}}{dt^{*}}=0$$

Therefore, we obtain the following system of PDE (given in matrix form, equation-2) in the main text):

$$\frac{dR}{dm_{1}}=\frac{\partial r}{\partial m_{1}}+\frac{\partial r}{\partial m_{2}}\cdot\frac{dm_{2}}{dm_{1}}+\frac{\partial r}{\partial\tau_{1}}\cdot\frac{d\tau_{1}}{dm_{1}}+\frac{\partial r}{\partial\tau_{2}}\cdot\frac{d\tau_{2}}{dm_{1}}+\frac{\partial r}{\partial\tau^{*}}\cdot\frac{d\tau^{*}}{dm_{1}}$$

$$\frac{dR}{dm_{2}}=\frac{\partial r}{\partial m_{2}}+\frac{\partial r}{\partial m_{1}}\cdot\frac{dm_{1}}{dm_{2}}+\frac{\partial r}{\partial\tau_{1}}\cdot\frac{d\tau_{1}}{dm_{2}}+\frac{\partial r}{\partial\tau_{2}}\cdot\frac{d\tau_{2}}{dm_{2}}+\frac{\partial r}{\partial\tau^{*}}\cdot\frac{d\tau^{*}}{dm_{2}}$$

$$\frac{dR}{dt_{1}}=\frac{\partial r}{\partial m_{1}}\frac{dm_{1}}{dt_{1}}+\frac{\partial r}{\partial m_{2}}\cdot\frac{dm_{2}}{dt_{1}}+\frac{\partial r}{\partial\tau_{1}}\cdot\frac{d\tau_{1}}{dt_{1}}+\frac{\partial r}{\partial\tau_{2}}\cdot\frac{d\tau_{2}}{dt_{1}}$$

$$\frac{dR}{dt_{2}}=\frac{\partial r}{\partial m_{1}}\frac{dm_{1}}{dt_{2}}+\frac{\partial r}{\partial m_{2}}\cdot\frac{dm_{2}}{dt_{2}}+\frac{\partial r}{\partial\tau_{1}}\cdot\frac{d\tau_{1}}{dt_{2}}+\frac{\partial r}{\partial\tau_{2}}\cdot\frac{d\tau_{2}}{dt_{2}}$$

$$\frac{dR}{dt^{*}}=\frac{\partial r}{\partial\tau^{*}}\frac{d\tau^{*}}{dt^{*}}$$

In an experiment where conditions are constant through time, *t_1_* and *t_2_* are constants and the system reduces to:

$$\frac{dR}{dm_{1}}=\frac{\partial r}{\partial m_{1}}+[\frac{\partial r}{\partial m_{2}}\cdot\frac{dm_{2}}{dm_{1}}]+\frac{\partial r}{\partial\tau^{*}}\cdot\frac{d\tau^{*}}{dm_{1}}$$

$$\frac{dR}{dm_{2}}=\frac{\partial r}{\partial m_{2}}+[\frac{\partial r}{\partial m_{1}}\cdot\frac{dm_{1}}{dm_{2}}]+\frac{\partial r}{\partial\tau^{*}}\cdot\frac{d\tau^{*}}{dm_{2}}$$

$$\frac{dR}{dt^{*}}=\frac{\partial r}{\partial m_{1}}\frac{dm_{1}}{dt^{*}}$$

In situations where the magnitude of the drivers are mutually independent, the terms brackets are zero.

1. **Experimental design**


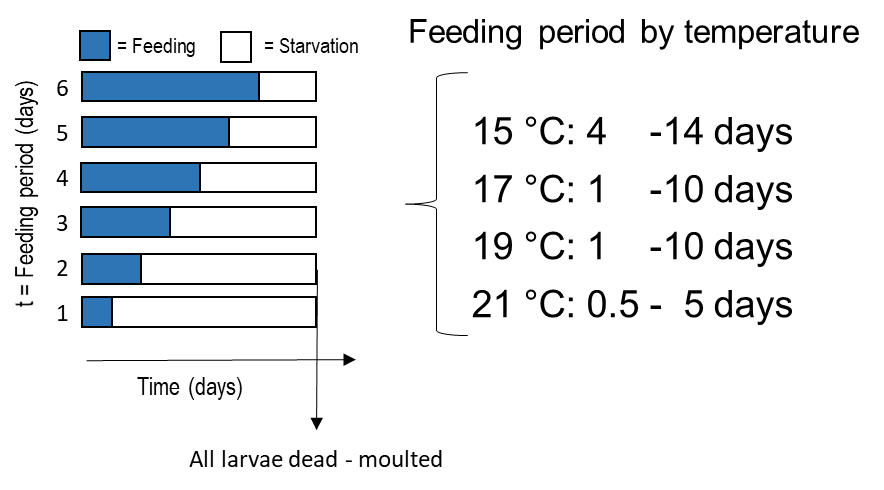


Supplementary Figure 1: Summary of PRS-experiment: Groups of larvae (e.g. from 1 to 6) were exposed to different initial feeding periods followed by a starvation period until larvae moulted or died; the experiment stopped when there were no remaining Zoea I larvae alive. Each such PRS experiment was repeated in parallel at four different temperatures with starvation period varying in order to compensate for the effect of temperature on developmental time.

1. **Supplementary tables**

Supplementary table 1. Parameter estimates for relationship between ln(developmental time) and ln(temperature).

| Parameter | Estimate | SE | t | P | Df | R^2^ |
| --- | --- | --- | --- | --- | --- | --- |
| Intercept | 9.45710 | 0.25361 | 37.29 | 2x10^-16^ | 87 | 0.9063 |
| Slope | -2.56929 | 0.08803 | -29.19 | 2x10^-16^ |  |  |

Supplementary Table 2. Summary of parameter estimates by temperature with starvation period in clock time by temperature. The fitted equation was the Boltzmann sigmodal: $R(t)=R_{M}+ \frac{R_{M}-R_{m}}{1+e^{-(t-t_{50})/k}}$ applied separately to each temperature condition. Data could not be fitted to a full model of four parameters, due to limited degrees of freedoms. Instead, it was fitted to a model assuming *Rm = 0* and *R_M_* = maximum survival obtained at each temperature. In addition, for 19 and 21 °C, the slope constant (k) showed very wide standard errors; hence, in that case, we ran a preliminary model with *k* as a free parameter, and then fitted a new model with *k* fixed in order to estimate *t_50._*

| Temperature °C | t_50_ | -95%CI | +95%CI | R^2^ | R_M_ | k |
| --- | --- | --- | --- | --- | --- | --- |
| 15 | 7.20 | 6.81 | 7.57 | 0.97 | 0.67 | 0.68 |
| 17 | 5.08 | 4.74 | 5.44 | 0.97 | 0.87 | 0.63 |
| 19 | 3.46 | 3.06 | 3.87 | 0.90 | 0.70 | 0.34 |
| 21 | 2.49 | 2.26 | 2.72 | 0.94 | 0.73 | 0.30 |

Supplementary Table 3. Parameter estimates for the effect of starvation periods on survival, scaled in biological time *τ*. The fitted equation was the Boltzmann sigmodal: $r(\tau)=r_{m}+ \frac{r_{M}-r_{m}}{1+e^{-(\tau-\tau_{50})/k}}$ ,where *r_m_* and *r_M_* are the asymptotic minimum and maximum survival respectively, *τ_50_* is the PRS_50_ in biological time (i.e. the time at which survival is 0.5 *r_M_*) and k is the slope parameter (e.g. sharp increases in survival reflect a small k).

| Parameter | Meaning | Estimate | SE | p<0.05 | R^2^ |
| --- | --- | --- | --- | --- | --- |
| *r_m_* | Minimum survival | 9.8 10^-3^ | 3.4 10^-2^ | No | 0.915 |
| *r_M_* | Maximum survival | 0.696 | 0.023 | Yes |  |
| *τ_50_* | PRS_50_ | 0.528 | 0.016 | Yes |  |
| k | Slope parameter | 0.052 | 0.012 | Yes |  |

Supplementary Table 4. Estimates of *τ_50_* (PRS_50_ in biological time) by temperature. Data for each temperature could not be fitted to a full model of four parameters as in Table S1, due to limited degrees of freedom. Instead it was fitted to a model assuming Min = 0 and Max = maximum survival obtained at each temperature. In addition, for 19 and 21 °C, the slope constant (k) showed very wide standard errors; hence, in that case, we ran a preliminary model with k as a free parameter, and then fitted a new model with k fixed in order to estimate *t_50._*

| Temperature °C | Estimate | -95%CI | +95%CI | R^2^ | r_M_ | k |
| --- | --- | --- | --- | --- | --- | --- |
| 15 | 0.58 | 0.55 | 0.61 | 0.97 | 0.67 | 0.055 |
| 17 | 0.58 | 0.54 | 0.62 | 0.97 | 0.87 | 0.072 |
| 19 | 0.52 | 0.48 | 0.56 | 0.95 | 0.70 | 0.053 |
| 21 | 0.47 | 0.43 | 0.51 | 0.94 | 0.73 | 0.057 |

Supplementary Table 5. Comparison of estimates of PRS_50_ expressed in biological time units (*τ_50_*) and clock units.

| Temperature °C | *τ_50_* | *t_50_* |
| --- | --- | --- |
| 15 | 0.58 | 7.2 |
| 17 | 0.58 | 5.1 |
| 19 | 0.52 | 3.5 |
| 21 | 0.47 | 2.4 |

1.
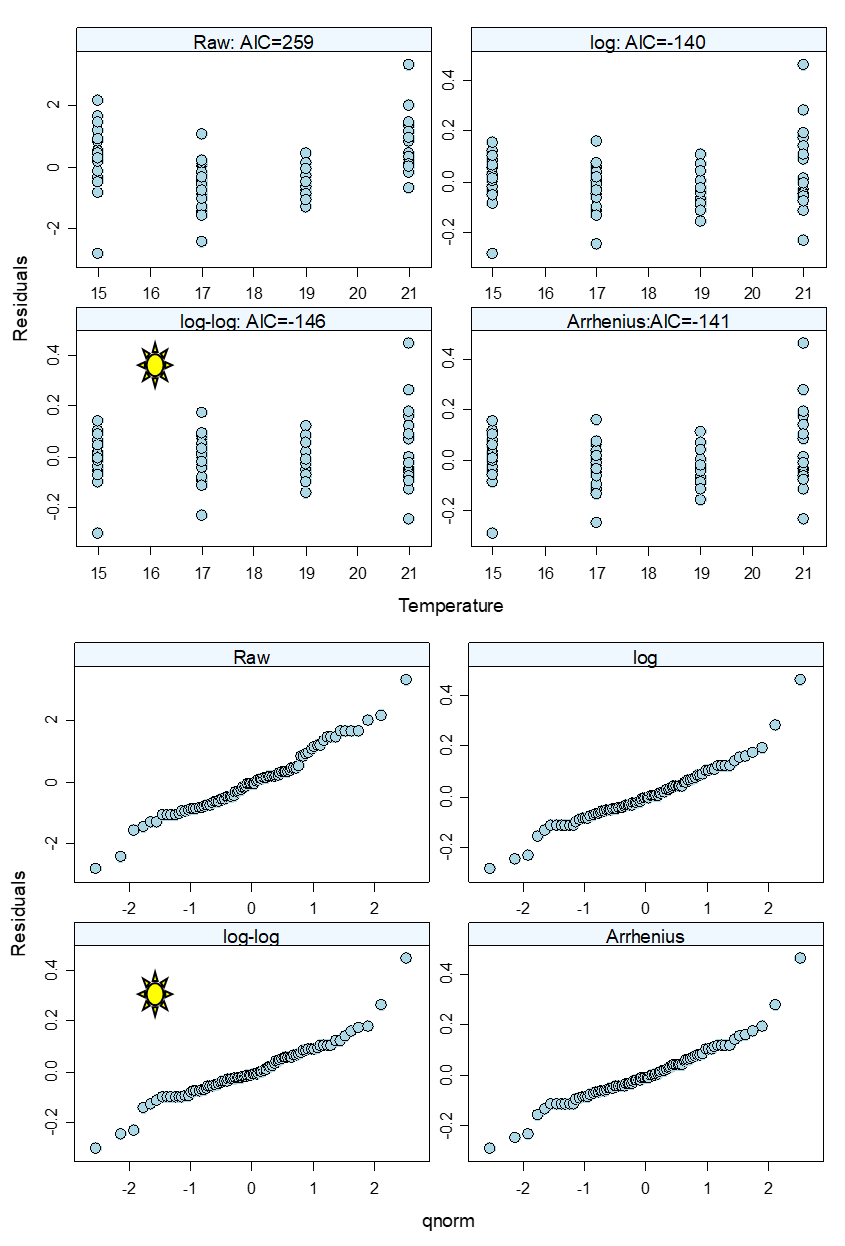
**Experimental results: case 3**

Supplementary Figure 2. Residuals obtained from model selection for the effect of temperature of developmental time. Top four panels: Residuals vs predictors; bottom panels: qqplots. Abbreviation of model names are as follows: Raw: Linear, log=exponential, log-log: power. AIC scores are given in the top panels. The best model (indicated by the star) was the power model (parameter estimates given in supplementary Table 1).

Supplementary Figure 3. Box-whisker plots of the relationship between developmental time and starvation period at different temperatures. Notice different y-scales and x-scales in the four plots. Rsq= R^2^ (determination coefficient), relationships were non-significant except for T=21 °C.

1. **Supplementary Code**

# A FRAMEWORK TO UNDERSTAND THE ROLE OF BIOLOGICAL TIME
# IN RESPONSES TO FLUCTUATING CLIMATE DRIVERS

# Luis Gimenez, Maria Noe Espinosa, Gabriela Torres

# Code by Luis Gimenez

# Case 1: Single driver response
#============================================================================

# 1. Libraries
library(plot3D)
library(rgl)

#--------------------------------------------------------------------------

# 2. Defining and storing coordinate predictors
mm<-seq(1,20,1) # Magnitude
tsc<-seq(1,20,1) # tsc = t in text = time scale of fluctuation
tast<-seq(1,20,1) # tast= t* in text = time of observation

#Note: we have t in the code to model more general cases
# However, this is not considered in figure 2: t will be fixed (line 41).

df<-data.frame(expand.grid(mm=mm, tsc=tsc, tast=tast))

#--------------------------------------------------------------------------

# 3. Calculation of developmental and biological time

df$DD<-1/df$mm
df$tausc<-df$tsc/df$DD
df$tauas<-df$tast/df$DD

#--------------------------------------------------------------------------

# 4. Calculation of response R
a=1
b=0.5
df$R<-df$mm*(a*df$tsc+b*df$tast)
# Note: the influence t and t* are additive – linear

#--------------------------------------------------------------------------

# 5. Figure 2: Image colour plot : mortality rate
XX<-mm
YY<-tast
dft<-subset(df, tsc==1) # plot will be done at a fixed t
ZZ<-matrix(dft$R, nrow=20)

iXX<-100/XX # Curve at fixed tau

# Plot of response
image2D(ZZ,XX,YY, xlab= "Magnitude", ylab="Time (clock): t*", resfac=3)

# Slice at fixed biological time of observation: tau*
points(XX, iXX, type="b",pch = 20, cex = 2, col = "yellow")
lines2D(seq(1,11,1), rep(12,11), lw=5,col= "black",add=TRUE)

# Slice at fixed clock time at observation t*
lines2D(seq(1,11,1), rep(12,11), lw=5,col= "white", lty="dashed" ,add=TRUE)
lines2D(seq(11,20,1), rep(12,10), lw=5,col= "white",add=TRUE)
lines2D(seq(11,20,1), rep(12,10), lw=5,col= "black", lty="dashed" ,add=TRUE)


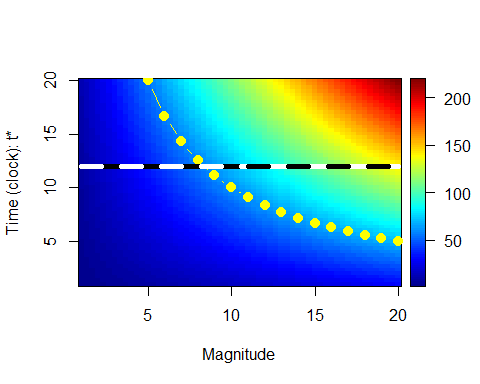


#################### END OF CODE ###########################################

**6.2 Case 2**

# A FRAMEWORK TO UNDERSTAND THE ROLE OF BIOLOGICAL TIME
# IN RESPONSES TO FLUCTUATING CLIMATE DRIVERS

# Luis Gimenez, Maria Noe Espinosa, Gabriela Torres

# Code by Luis Gimenez

# Case 2: Multiple driver interactions
#======================================================================

# 1. Libraries
library(lattice)
library(latticeExtra)
library(plot3D)
library(rgl)

#--------------------------------------------------------------------

# 2. Definition of coordinate predictors and storage in data.frame

Te<-seq(15,25,0.5) # Temperature = m1
S<-seq(15,35,0.5) # Second variable = m2
t1<-seq(1,40,0.5) # Clock time = t*
df<-data.frame(expand.grid(T=Te, S=S, t=t1))

# Predictors ranges are arbitrary; temperature is simulated between 0 and 25C

#--------------------------------------------------------------------

# 3. Parameters and equation of developmental time (O'Connor et al. 2007)

Ea=0.64 # activation energy
k=8.617333262145*10^-5 # Boltzmann constant
A=Ea/k
D0=exp(-22.47)
df$DD<-D0*exp(A/(df$T+273)) # Arrhenius function

#--------------------------------------------------------------------

# 4. Biological time: tau=τ*

df$tau<-df$t/df$DD

#--------------------------------------------------------------------

# 5. Function of survival in response to drivers

# Temperature: this code enables modelling of more general cases
# But here beta = 0 and there is no effect of temperature on survival

beta=0
df$fT=beta*df$T

# Second driver: We use a logistic function (sigmoid pattern)
# The function has 3 parameters: mu, alfa and inflection at S=25.
# Parameter values ensure the full range of survival within temperature 0-25C

alfa=0.1
mu=0.5
df$fs=alfa/(1+exp(mu*(df$S-25)))

# Response function (=R) of temperature and second driver
# R is an exponential decaying function of clock time with instantaneous rate # driven by the logistic function of the second driver

df$ff=(df$fs+df$fT)*df$t
df$R<-exp(-df$ff)

#--------------------------------------------------------------------

# 6. Calculations for figures

# Storing survival data at an arbitrary clock time
dft<-subset(df, t==40)

# Arbitrary biological time at tau* ~1
# Note the following: We do not have exact values of tau=1
# for each combination of predictors
# because tau is calculated from the equations it is not always exactly 1
# therefore we "slice" the response at values as near tau=1 as possible.
dfr<-subset(df, tau>1)
dfr2<-merge(aggregate(tau ~ S+T, FUN=min, data = dfr), dfr)

# Figure 3a: images in plot3D-------------

XX<-Te
YY<-S
ZZ<-matrix(dft$R, nrow=21) # clock time
ZZr<-matrix(dfr2$R, nrow=21) # biological time
par(mfrow = c(1, 2), mar=c(5.1, 3, 4.1, 2.1))
image2D(ZZ,XX,YY, xlab= "Temperature", ylab="Driver-2", resfac=3)
image2D(ZZr,XX,YY, xlab= "Temperature", ylab="Driver-2", resfac=3)

# Additional designs in figure 3 were made in powerpoint


# Figure 3b profile plots as if it were plots of means

# Choose a fixed set of predictor values
dfa<-subset(dft, S==15 | S==20| S==25| S==30 |S==35)
dfa<-subset(dfa, T==15 | T==18| T==21| T==24)

dfb<-subset(dfr2, S==15 | S==20| S==25| S==30 |S==35)
dfb<-subset(dfb, T==15 | T==18| T==21| T==24)

# Figure through package Lattice
mycols<-c( "red","orange", "light green", "light cyan","light blue" )
mykey<-list(title= "Driver 2", x=0.85, y=0.4,
 points= list(pch=21, cex=1.3, col="black", fill=mycols),
 text = list(c("15","20","25","30","35")))

mycols2<-c( "light blue", "light cyan", "orange","red")
mykey2<-list(x=0.1, y=0.9,
 points= list(pch=21, cex=1.3, col="black", fill=mycols2),
 text = list(c("15","18","21","24")))

# Pattern in clock time
ctime<-xyplot(R~S,groups=factor(T), pch=21, cex=1.3,data=dfa,type="b",lty=2,
 col="black", fill=mycols2, key = mykey2, ylim=c(0,1))

# Pattern in biological time
biotime<-xyplot(R~S,groups=factor(T), pch=21, cex=1.3,data=dfb, type="b",lty=2,
 col="black", fill=mycols2, key = mykey2,ylim=c(0,1))

c(ctime,biotime, x.same=FALSE, y.same=FALSE)


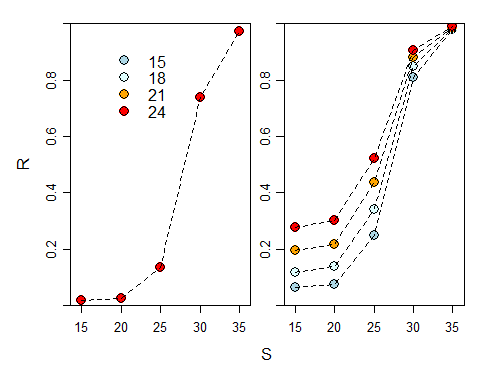


####################### END OF CODE#######################################

1. **Supplementary note 2: Derivation of equation of degree days**

Here we show that when phenology is driven by degree days, the equation of biological time scale of the fluctuation (*= τ*) falls within case 3a or 3b of the PDEs (equation 2 in main text) where *dτ /dt = L* and hence, *τ* is proportional to *m∙t* (m= magnitude of fluctuation, t: clock time of fluctuation, L = in the case of phenology, inverse of developmental time to a target life history event). The case 3c, where *dτ /dt = L+t dL/dt* requires that the developmental time has *t* explicitly in the equation.

We use equations given in Bonhomme (2000), page 3 and we define G = rate of development, *T_d_* = temperature, *T_h_*= thermal threshold, *a* = constant.

$G=a(T_{d}-T_{h})$ S2.1

The path of development defined as:

$1=a\sum_{0}^{n} (T_{d}-T_{h})\Delta t$ S2.2

We then note that the path of development defines the condition of *τ* =1 because *τ* is the fraction of time needed to complete the development to a specific stage.

We now introduce the clock time *(= x*). In all cases below the initial time is set to *x=0*, so that the time scale a fluctuation is defined as *t*. We can therefore express *τ* as follows:

$$\tau=\frac{a\sum_{0}^{t} (T_{d}-T_{h})\Delta t}{a[\sum_{0}^{t} \left( T_{d}-T_{h} \right)\Delta t+\sum_{t+1}^{n} \left( T_{d}-T_{h} \right)\Delta t]}$$

The denominator is just equation S2.2 divided into two fractions: the one associated to the time scale of the fluctuation and the remaining fraction needed to complete development. Because the denominator is = 1(see equation S2.2) we obtain:

$$\tau=a\sum_{0}^{t} (T_{d}-T_{h})\Delta t$$

In the following, we present several cases where we obtain an explicit expression for *τ*, setting *T_h_=0* for simplicity.

Case1: We assume that the fluctuation can be represented as a square wave of *period = t* and constant *temperature = m* in line with the PDEs of the main text (Figure A1). Under such condition, we obtain:

$$\tau=a\sum_{0}^{t} m \Delta t=amt$$

Because *τ = t∙L,* we identify *L* with *a∙m*, i.e. *L* is proportional to the magnitude of the fluctuation.


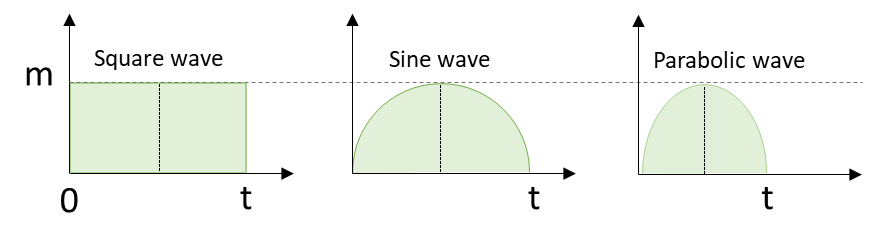


Supplementary Figure 4. Environmental fluctuations approximated as simple mathematical function in order to illustrate that biological time is proportional to the product mt in the case of phenology driver by degree days (m=magnitude of fluctuation, t= time scale of fluctuation).

We now explore more general cases, by defining time as a continuous variable. Equation 2 converts to

$1=a\int_{0}^{c} f\left( x \right)dx$ S2.3

where *f(x)* is a function describing the fluctuation in temperature over time. As in the discrete case, we define *τ* as:

$\tau=a\int_{0}^{t} f\left( x \right)dx$ S2.4

Case 2: we explore again a square wave as in case 1: In such case, *f(x)=m* and the solution to the integral gives *t∙m* in consistence with the discrete case.

Case 3: Fluctuation as a sine wave (Figure A1) of *amplitude = m* and *period = 2t*.

$\tau=a\int_{0}^{t} m\cdot sin(\frac{2\pi x}{2t})dx$ S2.4

Integrating by substitution and noting that cos(π)=-1 and cos(0)=1 we obtain and expression of *τ* proportional to *m∙t*

$\tau=-\frac{amt{[cos \left( \frac{\pi x}{t} \right)]}_{0}^{t}}{2\pi}=amt/\pi$ S2.5

In situations where fluctuations are approximated as the sum sine and cosine functions, the resulting *τ* will be also proportional to *m∙t* because the integral of the sum of trigonometric functions will be the sum of the integrals of each separated function.

Case 4: The fluctuation is approximated as a parabola. In such case we define the parabola as *f(x) = x(t-x)* so that *f=0* in *x=0* and *x=t,* meeting the requisites of the fluctuations considered here*.* In addition, the maximum of *f(x)* (= *fmax*) defines the amplitude of the fluctuation = m. By derivation we find the *fmax* occurs at *x=t/2*, and therefore *m=(t/2)^2^*. The sequence of steps below shows again that *τ* is proportional to *t∙m*.

$\tau=a\int_{0}^{t} x(t-x)dx$

$\tau=a[t\int_{0}^{t} x dx-\int_{0}^{t} x^{2}dx]$

$\tau=a[t\left| \frac{x^{2}}{2} \right|_{0}^{t}-\left| \frac{x^{3}}{3} \right|_{0}^{t}]$

$\tau=a[\frac{t^{3}}{2}-\frac{t^{3}}{3}]$

$\tau=at[\frac{{3t}^{2}-{2t}^{2}}{6}]$

$\tau=at[\frac{t^{2}}{6}]$

$\tau=at[\frac{2m}{3}]$

Reference: Bonhomme, R. Bases and limits to using “degree.day” units. *Europ. J. Agronomy* **13,**1-10 (2000).
